# Letter on “Synbiotics modulate gut microbiota and reduce enteritis and ventilator-associated pneumonia in patients with sepsis: a randomized controlled trial”

**DOI:** 10.1186/s13054-019-2319-7

**Published:** 2019-02-19

**Authors:** Alexander Reisinger, Vanessa Stadlbauer

**Affiliations:** 1 Intensive Care Unit, Department of Internal Medicine, Medical University Graz, Graz, Austria; 2 Division of Gastroenterology and Hepatology, Department of Internal Medicine, Medical University Graz, Graz, Austria

Recently a very interesting article was published by Shimizu et al. [1] about the use of synbiotic (pro- and prebiotic) therapy in patients with sepsis in the intensive care unit. We were looking forward to this randomized trial, because conclusive and high evidence data is still sparse on this topic in intensive care medicine. However, we believe that the paper has some methodological flaws that impair the validity of the study:

First, the study was performed single blinded to the participants (who were severely ill and ventilated at the time of study entry) but not blinded to the treating physicians and was not compared to placebo, therefore bearing a high risk of performance bias [2].

Second, the randomization was done in a 1:1 ratio with permutation blocks, but the allocation sequence was generated by the corresponding author of the study without further clarification. This uncertainty is not meeting good clinical practice standards, and in our opinion, automatized generation of randomization sequences should be used.

Third, in the methods of the study, the authors define that patients receiving other probiotics, or were expected to be discharged or transferred out of the ICU within 3 days after admission, were excluded. However, of the 127 patients screened for eligibility, 50 patients were excluded because they received probiotic therapy before inclusion or were “too severely ill to survive,” which in our understanding does not match the exclusion criterion “expected to be discharged or transferred out of the ICU.” In the study, it is not presented how the intensivists defined the severity of the disease and the probability of survival and the consecutive exclusion from the study.

Fourth, the analysis of the microbiota was done with rectal swabs in this study. As previous studies have shown that the microbiota present in the rectal swabs differs strongly from colonic lumen or mucosal samples collected by colonoscopy, rectal swabs may be not a true representation of the colonic microbiota especially in critically ill patients [3]. We are aware that a full colonoscopy in critically ill patients is not feasible, but retrieving samples by rectoscopy could be a reasonable compromise. Furthermore, the study samples were analyzed using a proprietary 16S- and 23S-PCR system (Yakult Intestinal Flora-SCAN (YIF-SCAN)), where a diagnostic advantage for the detection of the study probiotics (Yakult BL Seichoyaku) cannot be excluded. Using full 16S rRNA microbiome sequencing would be a more objective analysis method.
