# Supplementary material for: Letter on “Synbiotics modulate gut microbiota and reduce enteritis and ventilator-associated pneumonia in patients with sepsis: a randomized controlled trial”
Source: Crit Care. 2019 Feb 19;23:56. doi: 10.1186/s13054-019-2319-7 (PMC6381717; doi:10.1186/s13054-019-2319-7)
Supplement: Related texts

# Reply: Authors’ response

**Authors:** Kentaro Shimizu

We thank Dr. Reisinger and Dr. Stadlbauer for their insightful comments. As they pointed out, this was a single-blinded study in which the physicians were not blinded because it was difficult to create a placebo of Oligomate (the prebiotics used), which contains 55% or more galacto-oligosaccharide and is manufactured by the action of enzymes on lactose [4]. We used a 1:1 ratio with permutation blocks, but we did not automate the generation of randomization. These are limitations of the present research, and we expect the next step will be a double-blinded study. In response to the third comment, excluded patients were as follows: severely ill patients who could not be started enteral nutrition within 3 days of admission and non-severely ill patients who were able to eat after extubation and did not require enteral nutrition. In this research, the blood lactate level (median (IQR)) was 3.2 (1.7–5.7) mmol/L, and we thought that enteral nutrition could be provided with caution in patients treated with adrenergic agents, in accordance with the current guidelines [5]. Because patients with feeding intolerance were reported to have a higher rate of bacteremia and mortality [6], we used synbiotics when enteral nutrition could be tolerated.

In response to the fourth question, we agree with the comment that “rectal swabs may not be a true presentation of the colonic microbiota.” However, our preliminary research reported that the percentages of *Bacteroidetes* and *Firmicutes* from swab samples showed significant serial dynamic changes, and an extreme imbalance was associated with prognosis in critically ill patients [7]. Swab samples may change and be representative of the current colonic microbiota in accordance with the clinical situation.

In addition, our study was a quantitative research study of the main subset of microbiota, and other whole bacteria were not evaluated as described in the limitations. However, many 16S rRNA microbiome research studies only include proportions, and not quantities, and so it is difficult to evaluate true changes in bacterial counts by assessing proportional changes alone. It was reported in critically ill patients that the number of obligate anaerobes was significantly decreased and that the decrease of obligate anaerobes and the increase of facultative aerobes were associated with bacteremia and mortality [8]. Assessment of both proportions and quantities in microbiome research should be included in further study.
